# Disruptions in Tiopronin therapy: impacts on clinical outcomes of pediatric cystinuria patients during the COVID-19 pandemic

**DOI:** 10.1007/s00240-025-01767-4

**Published:** 2025-05-30

**Authors:** Hülya Gözde Önal, Hülya Nalçacioğlu, Demet Tekcan Karali, Meltem Necibe Ceyhan Bilgici, Ozlem Aydog, Ozan Özkaya, Ender Özdem, Saban Sarikaya

**Affiliations:** 1 Department of Pediatric Nephrology, Ondokuz Mayis University, Samsun, Turkey; 2 Department of Radiology, Ondokuz Mayis University, Samsun, Turkey; 3 Medical Park Bahcelievler Hospital, Pediatric Nephrology Clinic, Istanbul, Turkey; 4 Liv Bahçeşehir Hospital, Department of Pediatric Nephrology, İstinye University, İstanbul, Turkey; 5 Department of Urology, Ondokuz Mayis University, Samsun, Turkey; 6 Present Address: Department of Pediatric Nephrology, Samsun Training and Research Hospital, Samsun, Turkey

**Keywords:** Cystinuria, Tiopronin, COVID-19, Renal function, Nephrolithiasis

## Abstract

Cystinuria, characterized by defective renal absorption of cystine causing recurrent nephrolithiasis, demands ongoing management. This study examines the effects of COVID-19-related disruptions in tiopronin availability on the clinical outcomes of pediatric cystinuria patients. This retrospective cohort study analyzed medical records of 11 pediatric patients with cystinuria, followed from 2001 to 2023. Patients were diagnosed using urine microscopy/biochemistry and stone composition analysis. Clinical outcomes, including renal function and stone status, were assessed using serial ultrasonographic evaluations and 24-hour urinary cystine measurements. At diagnosis, the median age was 13 months, and 63.6% were female. Acute kidney injury was observed in 36.4% of patients, with 27.3% requiring emergency dialysis. The interruption of tiopronin treatment led to significant renal function deterioration and increased stone burden, as evidenced by an increase in the median number of kidney stones from 2 (IQR: 1–3) to 4 (IQR: 2–5, *p* = 0.045) and a rise in 24-hour urinary cystine levels from 286 mg/1.73 m² (IQR: 82–552.5) to 434 mg/1.73 m² (IQR: 198–854, *p* = 0.043). Data prior to the interruption showed a median glomerular filtration rate (GFR) of 80.4 mL/min/1.73 m² and creatinine levels of 2.47 mg/dL. After resuming tiopronin, there was a notable improvement to a median GFR of 161 mL/min/1.73 m² and creatinine levels of 0.48 mg/dL. Managing cystinuria during the pandemic underscored the critical role of continuous access to medications like tiopronin in preventing renal deterioration. Developing strategies to ensure an uninterrupted drug supply during global health emergencies is crucial for managing chronic conditions such as cystinuria.

**Supplementary Information:**

The online version contains supplementary material available at 10.1007/s00240-025-01767-4.

## Introduction

Cystinuria is a genetic disorder that often leads to kidney stone formation in children, primarily due to the kidneys’ impaired ability to reabsorb the amino acid cystine—a result of cystine carrier malfunctions. Cystine’s low solubility in urine predisposes individuals to stone development [1, 2]. The condition is relatively rare, occurring in approximately one out of every 7,000 births globally, and is a significant contributor to pediatric nephrolithiasis [3]. Early and effective intervention is critical for preventing stone formation and maintaining renal health. Treatment strategies for cystinuria encompass a comprehensive approach aimed at halting disease progression and minimizing the risk of stone formation, including substantial fluid intake, dietary adjustments, and urine alkalization [4–6]. Pharmacotherapy is also vital, with tiopronin playing a pivotal role by converting cystine into more soluble forms, thus reducing urinary cystine levels and substantially diminishing the risk of stone formation [7–9]. The efficacy of tiopronin hinges on consistent administration, which can be compromised during public health crises like pandemics, leading to interruptions in treatment and adverse health outcomes.

Initiated in March 2020, the COVID-19 pandemic has severely restricted access to healthcare services worldwide, negatively influencing the management of chronic conditions, including cystinuria [10, 11]. Patients dependent on tiopronin faced significant access challenges, resulting in management disruptions and detrimental clinical outcomes.

This study aims to explore the impact of pandemic-related interruptions in tiopronin access on the clinical outcomes of pediatric patients with cystinuria.

## Methods

This retrospective cohort study was conducted on 11 pediatric patients diagnosed with cystinuria at the Pediatric Nephrology Unit of Ondokuz Mayıs University Hospital, spanning from January 2001 to January 2023. This study was performed in line with the principles of the Declaration of Helsinki. The local ethics committee granted approval for this study (decision no: B.30.2.ODM.0.20.08/227). Informed consent was obtained from all participants or their legal guardians prior to their inclusion in the study.

The diagnosis of cystinuria was established through urine microscopy/biochemistry and stone composition analysis [12]. At diagnosis, patient data such as age, gender, symptoms at presentation, acute kidney failure, dialysis requirements, the presence of kidney stones, familial stone history, and consanguinity were systematically recorded. Key clinical metrics, including glomerular filtration rate (GFR), serum creatinine levels, spot urine cystine/creatinine ratio, and 24-hour urine cystine levels were measured at diagnosis and monitored over time. Serum creatinine values were obtained during routine outpatient follow-ups, except for patients requiring hospitalization, where values were collected during inpatient admissions. The timing of these measurements was standardized to minimize potential bias introduced by variations in care during the pandemic. The GFR was calculated using the Schwartz formula: GFR (mL/min/1.73 m²) = (k × height in cm)/serum creatinine in mg/dL, where k is a constant based on age and sex. The same formula was consistently applied at diagnosis and during follow-up assessments. Detailed documentation of the treatment regimens and interventions, including urological procedures, was maintained. Standard therapeutic protocols involving fluid consumption and dietary limitations were implemented for all patients, who also received tiopronin and potassium citrate therapy.

The COVID-19 pandemic was officially recognized in Türkiye on March 11, 2020, by the Ministry of Health [13]. From March 2022 to December 2022, during the pandemic, the study subjects experienced an interruption in their tiopronin therapy due to supply chain disruptions. This interruption was complete, with no tiopronin available to the patients during this period. This break in treatment allowed for the comparative evaluation of clinical parameters such as creatinine, GFR, spot urine cystine/creatinine ratio, and 24-hour urine cystine levels between the final clinic visit before and the first clinic visit after the resumption of medication availability.

### Statistics

Statistical analyses were conducted using IBM’s SPSS software, version 29 (IBM Corp., Armonk, NY). Descriptive statistics summarized the data, presenting counts and percentages for categorical variables, and either means with standard deviations or medians with interquartile ranges (25 th − 75 th percentiles) for continuous variables. We assessed the assumption of normal distribution for each group by examining histograms and applying the Shapiro-Wilk test. Comparisons within dependent groups were made using suitable statistical tests, including the paired t-test or the Wilcoxon or sign tests for continuous variables. 95% confidence intervals (CIs) for paired differences were calculated using the standard error of the mean difference for t-tests or based on the rank-sum distribution for non-parametric tests. All tests were two-tailed, with a significance threshold set at *p* < 0.05, indicating statistical significance. To minimize Type 1 error, Bonferroni correction was applied to the remaining comparisons.

## Results

This study involved a cohort of 11 patients, with their diagnostic characteristics summarized in Table 1. The median age at diagnosis was 13 (range 4–150) months, with a predominance of female patients, who constituted 63.6% (*n* = 7) of the sample. Over half of the patients (54.5%, *n* = 6) reported dysuria as their primary complaint, while nearly half (45.5%, *n* = 5) were diagnosed with urinary tract infections at their initial evaluation. Acute kidney injury was noted in 36.4% (*n* = 4) of the cases at diagnosis, and 27.3% (*n* = 3) required immediate dialysis. In patients who presented with postrenal obstruction, baseline serum creatinine and GFR values were obtained within 48 h following relief of obstruction and after dialysis, if performed. For the remaining patients, initial values reflected outpatient measurements prior to the initiation of tiopronin therapy. Diagnostic ultrasonography identified a median of three stones (IQR 1–2) in the right kidney and one stone (IQR 0–2) in the left, with bilateral kidney stones observed in 63.6% of the patients (*n* = 7). A noTable 36.4% of the patients (*n* = 4) had a family history of kidney stones, with an equivalent percentage reporting consanguinity.

Metabolic profiles at diagnosis indicated an average GFR of 100.7 ± 37.8 mL/min/1.73 m² and creatinine levels of 0.53 ± 0.18 mg/dL. The median spot urine cystine-to-creatinine ratio was 831 (IQR 338–1230.5), and the 24-hour urine cystine concentration was 615 mg/1.73 m² (IQR 392.75–846.25). Further details on urine analyses are available in Table 2.

**Table 1:** Initial diagnostic characteristics of pediatric cystinuria patients

Category	Detail	Patients (*n* = 11)
Demographic Information	Age (in months, IQR_1_)	13 (range 4–150)
	Sex (Female)	7 (63.6%)
Symptoms and Findings	Dysuria	6 (54.5%)
	Urinary Tract Infections	5 (45.5%)
Renal Complications	Acute Kidney Injury	4 (36.4%)
	Dialysis Indication	3 (27.3%)
Kidney Stone Details	Right Kidney	3 (IQR_1_ 1–3)
	Left Kidney	1 (IQR_1_ 0–4)
	Bilateral Stones	7 (63.6%)
Family Medical History	Nephrolithiasis	4 (36.4%)
Parental Marital Status	Consanguineous Marriages	4 (36.4%)

_1_ Interquartile range

**Table 2:** Metabolic profiles at initial diagnosis of pediatric cystinuria patients

Variables	Patients (*n* = 11)
Glomerular filtration rate (mL/min/1.73 m²)	100.7 ± 37.8
Creatinine (mg/dL)	0.53 ± 0.18
Spot urine cysteine to creatine ratio	831 (IQR_1_ 338–1230.5)
24-hour urine cysteine (mg/1,73 m^2^/g)	615 (IQR_1_ 392.75–846.25)
Spot urine citrate to creatine ratio	0.83 (IQR_1_ 0.58–1.53)
24-hour urine citrate (mg/1,73 m^2^/g)	259 (IQR_1_ 218.5–353.5)
Spot urine calcium to creatine ratio	0.09 (IQR_1_ 0.03–0.15)
24-hour urine calcium (mg kg/day)	2 (IQR_1_ 1.4–3.8)
24-hour urine uric acid (mg/1,73 m^2^/g)	498 (IQR_1_ 241.25–662.75)
Spot urine oxalate to creatine ratio	0.03 (IQR_1_ 0.025–0.41)
24-hour urine oxalate (mmol/1,73 m^2^/g)	0.5 (IQR_1_ 0.43–0.8)

_1_ Interquartile range

Follow-up data, presented in Table 3, revealed an average monitoring period of 7.1 ± 5 years. The most recent creatinine levels during treatment averaged 0.48 ± 0.22 mg/dL, with a last recorded GFR of 161 ± 77.1 mL/min/1.73 m². Throughout the follow-up, 9.1% of patients (*n* = 1) received extracorporeal shock wave lithotripsy (ESWL), 36.4% (*n* = 4) underwent ureteroscopy (URS), and 72.7% (*n* = 8) were treated with percutaneous nephrolithotomy (PNL). All participants consistently received tiopronin and potassium citrate treatments.

**Table 3:** Summary of Follow-Up outcomes

Follow-Up Metrics	Data (*n* = 11)
Duration of Follow-up (years)	7.1 ± 5
Creatinine Levels at Last Follow-up (mg/dl)	0.48 ± 0.22
GFR_1_ at Last Follow-up (mL/min/1.73 m²)	161 ± 77.1
Patients undergoing ESWL_2_	9.1% (1)
Patients undergoing URS_3_	36.4% (4)
Patients undergoing PNL_4_	72.7% (8)
Patients receiving potassium citrate	100% (11)
Patients receiving tiopronin	100% (11)

_1_ Glomerular Filtration Rate

_2_ Extracorporeal shock wave lithotripsy

_3_ Ureteroscopy

_4_ Percutaneous nephrolithotomy

After treatment interruption, average creatinine levels increased slightly (0.58 ± 0.24 mg/dL compared to 0.48 ± 0.22 mg/dL during treatment), showing a mean increase of 0.1 mg/dL (95% CI 0.02–0.18, *p* = 0.024). Additionally, three patients demonstrated an absolute increase in serum creatinine of more than 0.3 mg/dL, and one patient exhibited a greater than 50% increase compared to treatment-phase values. After treatment interruption, GFR (152.5 ± 78.5 mL/min/1.73 m²) decreased by 8.7 mL/min/1.73 m² (95% CI 4.3–13.2, *p* = 0.001). Evaluations following treatment interruption also showed significant increases in both the median spot urine cystine-to-creatinine ratio and 24-hour urinary cystine levels compared to levels during treatment (*p* = 0.045 and *p* = 0.043, respectively) (Table 4). Furthermore, the interruption of tiopronin therapy was associated with a significant increase in stone burden. The median number of kidney stones detected on follow-up ultrasonography rose from 2 (IQR: 1–3) during treatment to 4 (IQR: 2–5) after cessation (*p* = 0.045), reflecting heightened lithogenic activity during the drug discontinuation period.

**Table 4:** Analysis of the impact of treatment interruption

Parameter	During Treatment	After cessation	*p*	Mean Difference (95% CI_1_)
Creatinine (mg/dL)	0.48 ± 0.22	0.58 ± 0.23	0.024	0.1 (0.02–0.18)
GFR_2_ (mL/min/1.73 m²)	161 ± 77.1	152.5 ± 78.5	0.001	8.7 (4.3–13.2)
Spot Urine Cystine-Creatinine Ratio	468 (IQR_3_ 210.5–811.5)	692.5 (IQR_3_ 320.75–1061.75)	0.045	-
24-hour Urinary Cystine (mg/1.73 m²)	286 (IQR_3_ 82–552.5)	434 (IQR_3_ 198–854)	0.043	-

_1_ Confidence interval

_2_ Glomerular Filtration Rate

_3_ IQR: Interquartile range

## Discussion

This research highlights the significant impact that interruptions in access to essential drugs like tiopronin, which is critical for the treatment of pediatric cystinuria patients, have had on renal functions during the COVID-19 pandemic. The pandemic-induced disruptions in tiopronin therapy resulted in notable increases in creatinine levels and significant reductions in GFR. The increase in serum creatinine levels during treatment interruption reflects a direct deterioration in renal function. In children, even small elevations in creatinine are significant due to their lower baseline renal reserve compared to adults. This underscores the sensitivity of pediatric kidneys to disruptions in therapy and highlights the critical importance of continuous tiopronin availability. The observed GFR decline of 8.7 mL/min/1.73 m², although statistically significant, is clinically concerning. The decrease in GFR observed during the tiopronin cessation period may be attributed to an increase in obstructive stone events. Without tiopronin, urinary cystine levels rise, leading to the formation and growth of cystine stones, which can obstruct urinary flow and cause acute kidney injury or chronic damage. There was also a marked rise in the levels of cystine in 24-hour urine samples. These findings underscore the detrimental effects that breaks in continuous pharmaceutical therapy can have on the health status of individuals with cystinuria.

Cystinuria manifests clinically as recurrent kidney stones, resulting from the defective reabsorption of specific amino acids within the renal tubules [14]. This condition is particularly concerning due to its potential to evolve into chronic kidney disease (CKD). Cystinuria is caused by mutations in the subunits of the rBAT and b^0,+^AT transporter proteins [15], which impair the reabsorption of cystine and other amino acids, thereby causing cystine to accumulate in the urine. The low solubility of cystine at physiological pH levels creates a favorable environment for stone formation [16].

Patients with cystinuria frequently present with a diverse array of clinical manifestations. In a case series from Türkiye, cystinuria was identified as the cause of acute kidney injury in three young children, who exhibited various nonspecific symptoms due to urinary tract obstructions [17]. Similarly, in this study, five patients were diagnosed with urinary tract infections, four suffered from acute kidney failure, and three required immediate dialysis at the time of diagnosis. Additionally, bilateral kidney stones were identified in seven patients. These findings highlight that individuals with cystinuria can display a range of clinical symptoms attributable to the recurrent nature of the stone burden. Such recurrent stone formation can lead to multiple surgical interventions throughout a patient’s life, potentially causing further decline in kidney function [18].

Cystinuria carries a heightened risk of CKD compared to other forms of nephrolithiasis, largely due to the recurrent stone formation and the blockages caused by the accumulation of cystine crystals [19]. These obstructions are prone to causing chronic inflammation and subsequent renal scarring. A deep understanding of cystinuria’s pathophysiology highlights the crucial need for meticulous monitoring and proactive management to mitigate stone formation and protect renal function. Present therapeutic approaches are centered on enhancing cystine solubility by alkalinizing the urine and promoting cystine elimination using strategies that increase urine volume and involve dietary adjustments [20].

The findings from this study highlight the vulnerability of pediatric cystinuria patients to treatment interruptions during global health crises like the COVID-19 pandemic. These interruptions reveal the delicate balance required to maintain renal function in chronic conditions and underline the necessity of systemic strategies to ensure continuous access to critical medications, even during emergencies. In this research, all participants were treated with tiopronin in addition to the standard protocols for cystinuria management. Given the potential adverse effects linked to tiopronin, such as membranous nephropathy and minimal change disease, regular monitoring of the urine protein/creatinine ratio is advised from the beginning of treatment and throughout its duration to facilitate early detection and management of these severe complications [21, 22]. The COVID-19 pandemic brought significant disruptions to tiopronin access, further highlighting the critical nature of such monitoring. Notably, during the periods when drug access was interrupted, there was a significant rise in the patients’ spot urine cystine/creatinine ratios compared to earlier measures. This observation underscores the negative impact that treatment interruptions can have on renal function and stresses the necessity of uninterrupted pharmaceutical therapy.

## Limitations

A primary limitation of this study is the small patient cohort. Additionally, the timing of serum creatinine measurements, which varied between routine outpatient follow-ups and inpatient admissions, may have introduced bias, particularly during the pandemic when healthcare access and care patterns were disrupted. Furthermore, the retrospective nature of the study may lead to incomplete data or insufficient evaluation of factors like adherence to treatment. These issues could potentially impact the study’s findings and restrict the generalizability of the results.

## Conclusion

This research underscores the necessity of uninterrupted access to crucial medications like tiopronin for maintaining renal function in cystinuria patients. The management complexities of chronic conditions during global health emergencies such as pandemics highlight the critical planning needs within healthcare systems. Thus, maintaining continuous treatment access, even in times of crisis, should be a top priority in managing pediatric cystinuria.

## Electronic supplementary material

Below is the link to the electronic supplementary material.

Supplementary Material 1

## Data Availability

No datasets were generated or analysed during the current study.

## References

[1] Ripa F, Pietropaolo A, Geraghty R, Griffin S, Cook P, Somani B (2023) Outcomes of paediatric cystine stone management: results of a systematic review. Curr Urol Rep 24(8):371–380. 10.1007/s11934-023-01162-9 37079195 10.1007/s11934-023-01162-9

[2] Mikel CC, Goldfarb DS, Ponte A, Steigelman K, Latyshev S (2022) Accurate 24-h urine cystine quantification for patients on cystine-binding thiol drugs. Urolithiasis 50(6):721–727. 10.1007/s00240-022-01364-9 36201021 10.1007/s00240-022-01364-9 PMC9584971

[3] Chillarón J, Font-Llitjós M, Fort J et al (2010) Pathophysiology and treatment of cystinuria. Nat Rev Nephrol 6(7):424–434. 10.1038/nrneph.2010.69 20517292 10.1038/nrneph.2010.69

[4] Kirsch-Noir F, Thomas J, Fompeydie D, Debré B, Zerbib M, Arvis G (2000) Lithiase cystinique: enseignements de L’étude D’une Série de 116 Cas [Cystine lithiasis: study of a series of 116 Cases]. Prog Urol 10(6):1135–1144 11217549

[5] Modersitzki F, Goldfarb DS, Goldstein RL, Sur RL, Penniston KL (2020) Assessment of health-related quality of life in patients with cystinuria on Tiopronin therapy. Urolithiasis 48:313–320. 10.1007/s00240-019-01174-6 31834425 10.1007/s00240-019-01174-6 PMC7335368

[6] Sakhaee K, Maalouf NM, Sinnott B (2012) Kidney stones 2012: pathogenesis, diagnosis, and management. J Clin Endocrinol Metabolism 97(6):1847–1860. 10.1210/jc.2011-3492 10.1210/jc.2011-3492 PMC3387413 22466339

[7] Modersitzki F, Goldfarb DS, Goldstein RL, Sur RL, Penniston KL (2020) Assessment of health-related quality of life in patients with cystinuria on Tiopronin therapy. Urolithiasis 48(4):313–320. 10.1007/s00240-019-01174-6 31834425 10.1007/s00240-019-01174-6 PMC7335368

[8] Micallef S, Delicata L, Agius JF, Holwill S (2022) Tiopronin-induced membranous nephropathy in a patient with refractory Familial cystinuria. Br J Hosp Med (Lond) 83(8):1–4. 10.12968/hmed.2022.0037 36066303 10.12968/hmed.2022.0037

[9] Servais A, Thomas K, Dello Strologo L et al (2021) Cystinuria: clinical practice recommendation. Kidney Int 99(1):48–58. 10.1016/j.kint.2020.06.035 32918941 10.1016/j.kint.2020.06.035

[10] Faiva E, Hashim HT, Ramadhan MA et al (2021) Drug supply shortage in Nigeria during COVID-19: efforts and challenges. J Pharm Policy Pract 14(1):17. 10.1186/s40545-021-00302-1 33482871 10.1186/s40545-021-00302-1 PMC7820524

[11] Uwizeyimana T, Hashim HT, Kabakambira JD et al (2021) Drug supply situation in Rwanda during COVID-19: issues, efforts and challenges. J Pharm Policy Pract 14(1):12. 10.1186/s40545-021-00301-2 33472702 10.1186/s40545-021-00301-2 PMC7816054

[12] Asi T, Dogan HS, Bozaci AC, Citamak B, Altan M, Tekgul S (2020) A single center’s experience in pediatric cystine stone disease management: what changed over time? Urolithiasis 48(6):493–499. 10.1007/s00240-020-01200-y 32556828 10.1007/s00240-020-01200-y

[13] Turkish Republic Ministry of Health (2020) Birincil Vaka. https://covid19.saglik.gov.tr/TR-66444/birincil‐vaka.html

[14] Sadiq S, Cil O (2022) Cystinuria: an overview of diagnosis and medical management. Turk Arch Pediatr 57(4):377–384. 10.5152/turkarchpediatr.2022.22105 35822468 10.5152/TurkArchPediatr.2022.22105 PMC9317473

[15] Palacín M, Nunes V, Font-Llitjós M et al (2005) The genetics of heteromeric amino acid transporters. Physiol (Bethesda) 20:112–124. 10.1152/physiol.00051.2004 10.1152/physiol.00051.2004 15772300

[16] D’Ambrosio V, Capolongo G, Goldfarb D, Gambaro G, Ferraro PM (2022) Cystinuria: an update on pathophysiology, genetics, and clinical management. Pediatr Nephrol 37(8):1705–1711. 10.1007/s00467-021-05342-y 34812923 10.1007/s00467-021-05342-y

[17] Nalcacioglu H, Ozden E, Genc G, Yakupoglu YK, Sarikaya S, Ozkaya O (2013) An uncommon cause of acute kidney injury in young children: cystinuria. J Pediatr Urol 9(1):e58–e63. 10.1016/j.jpurol.2012.08.006 23099233 10.1016/j.jpurol.2012.08.006

[18] Dello Strologo L, Pras E, Pontesilli C et al (2002) Comparison between SLC3A1 and SLC7A9 cystinuria patients and carriers: a need for a new classification. J Am Soc Nephrol 13(10):2547–2553. 10.1097/01.asn.0000029586.17680.e5 12239244 10.1097/01.asn.0000029586.17680.e5

[19] Rhodes HL, Yarram-Smith L, Rice SJ et al (2015) Clinical and genetic analysis of patients with cystinuria in the united Kingdom. Clin J Am Soc Nephrol 10(7):1235–1245. 10.2215/cjn.10981114 25964309 10.2215/CJN.10981114 PMC4491297

[20] Prot-Bertoye C, Lebbah S, Daudon M et al (2019) Adverse events associated with currently used medical treatments for cystinuria and treatment goals: results from a series of 442 patients in France. BJU Int 124(5):849–861. 10.1111/bju.14721 30801923 10.1111/bju.14721

[21] Zhong H, Wang L, Gu ZC, Cui M, Liu XY, Pang XY (2019) Minimal change disease induced by Tiopronin: a rare case report and a review of the literature. Ann Transl Med 7(16):398. 10.21037/atm.2019.07.42 31555712 10.21037/atm.2019.07.42 PMC6736807

[22] Eisner BH, Goldfarb DS, Baum MA et al (2020) Evaluation and medical management of patients with cystine nephrolithiasis: A consensus statement. J Endourol 34(11):1103–1110. 10.1089/end.2019.0703 32066273 10.1089/end.2019.0703 PMC7869875

