# Correction: Disruptions in Tiopronin therapy: impacts on clinical outcomes of pediatric cystinuria patients during the COVID-19 pandemic

**DOI:** 10.1007/s00240-025-01802-4

**Published:** 2025-06-25

**Authors:** Hülya Gözde Önal, Hülya Nalçacioğlu, Demet Tekcan Karali, Meltem Necibe Ceyhan Bilgici, Ozlem Aydog, Ozan Özkaya, Ender Özden, Saban Sarikaya

**Affiliations:** 1 Department of Pediatric Nephrology, Ondokuz Mayis University, Samsun, Turkey; 2 Department of Radiology, Ondokuz Mayis University, Samsun, Turkey; 3 Medical Park Bahcelievler Hospital, Pediatric Nephrology Clinic, Istanbul, Turkey; 4 Liv Bahçeşehir Hospital, Department of Pediatric Nephrology, İstinye University, Istanbul, Turkey; 5 Department of Urology, Ondokuz Mayis University, Samsun, Turkey; 6 Present address: Department of Pediatric Nephrology, Samsun Training and Research Hospital, Samsun, Turkey

**Correction: Urolithiasis (2025) 53:103** 10.1007/s00240-025-01767-4

In the original version of this article, the author’s name Ender Özden was incorrectly written as Ender Özdem.

