# Supplementary material for: Disruptions in Tiopronin therapy: impacts on clinical outcomes of pediatric cystinuria patients during the COVID-19 pandemic
Source: Urolithiasis. 2025 May 30;53(1):103. doi: 10.1007/s00240-025-01767-4 (PMC12125051; doi:10.1007/s00240-025-01767-4)
Supplement: Supplementary file 1 — Supplementary Material 1 [file 240_2025_1767_MOESM1_ESM.docx]

Supplementary Table 1. Individual Patient Data

| Patient | Diagnosis Age (months) | Follow-up (months) | Initial Creatinine (mg/dL) | Treatment Creatinine (mg/dL) | Cessation Creatinine (mg/dL) | Initial GFR (mL/min/1.73 m²) | Treatment GFR (mL/min/1.73 m²) | Cessation GFR (mL/min/1.73 m²) |
| --- | --- | --- | --- | --- | --- | --- | --- | --- |
| P1 | 13 | 131 | 6.35 | 0.39 | 0.49 | 6.7 | 117 | 117 |
| P2 | 150 | 18 | 0.61 | 0.56 | 0.66 | 130 | 178 | 171 |
| P3 | 12 | 120 | 3.57 | 0.17 | 0.27 | 11 | 131 | 121 |
| P4 | 12 | 204 | 4.20 | 0.74 | 0.84 | 6 | 134 | 127 |
| P5 | 54 | 90 | 0.40 | 0.54 | 0.64 | 137 | 161 | 158 |
| P6 | 4 | 20 | 0.32 | 0.35 | 0.45 | 155 | 372 | 365 |
| P7 | 27 | 105 | 6.80 | 0.55 | 0.65 | 7.2 | 198 | 184 |
| P8 | 6 | 126 | 0.27 | 0.37 | 0.47 | 146 | 117 | 105 |
| P9 | 120 | 60 | 43 | 10 | 1.10 | 14 | 103 | 83 |
| P10 | 6 | 12 | 0.22 | 0.26 | 0.36 | 160 | 163 | 155 |
| P11 | 35 | 46 | 0.35 | 0.40 | 0.50 | 112 | 95 | 87 |
